# Supplementary figures and images for: Spatial benthic community analysis of shallow coral reefs to support coastal management in Culebra Island, Puerto Rico
Source: PeerJ. 2020 Oct 14;8:e10080. doi: 10.7717/peerj.10080 (PMC7568481; doi:10.7717/peerj.10080)

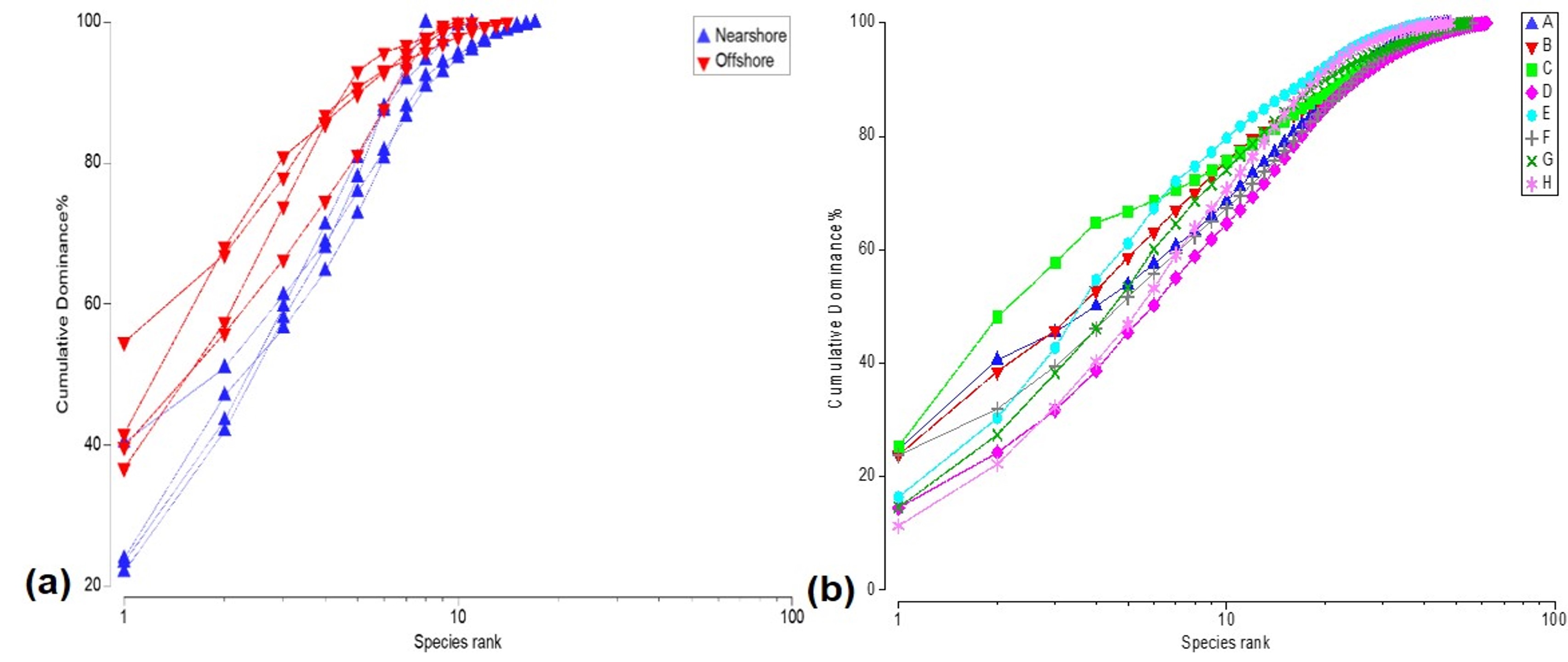

Supplement: Supplemental Information 1 — (A) % Cover of scleractinian and hydrocoral species between distance zones and (B) % Scleractinian cover among localities. Offshore locations exhibit higher coral cover, but inshore locality C stands out for its scleractinian dominance. [file peerj-08-10080-s001.jpg]

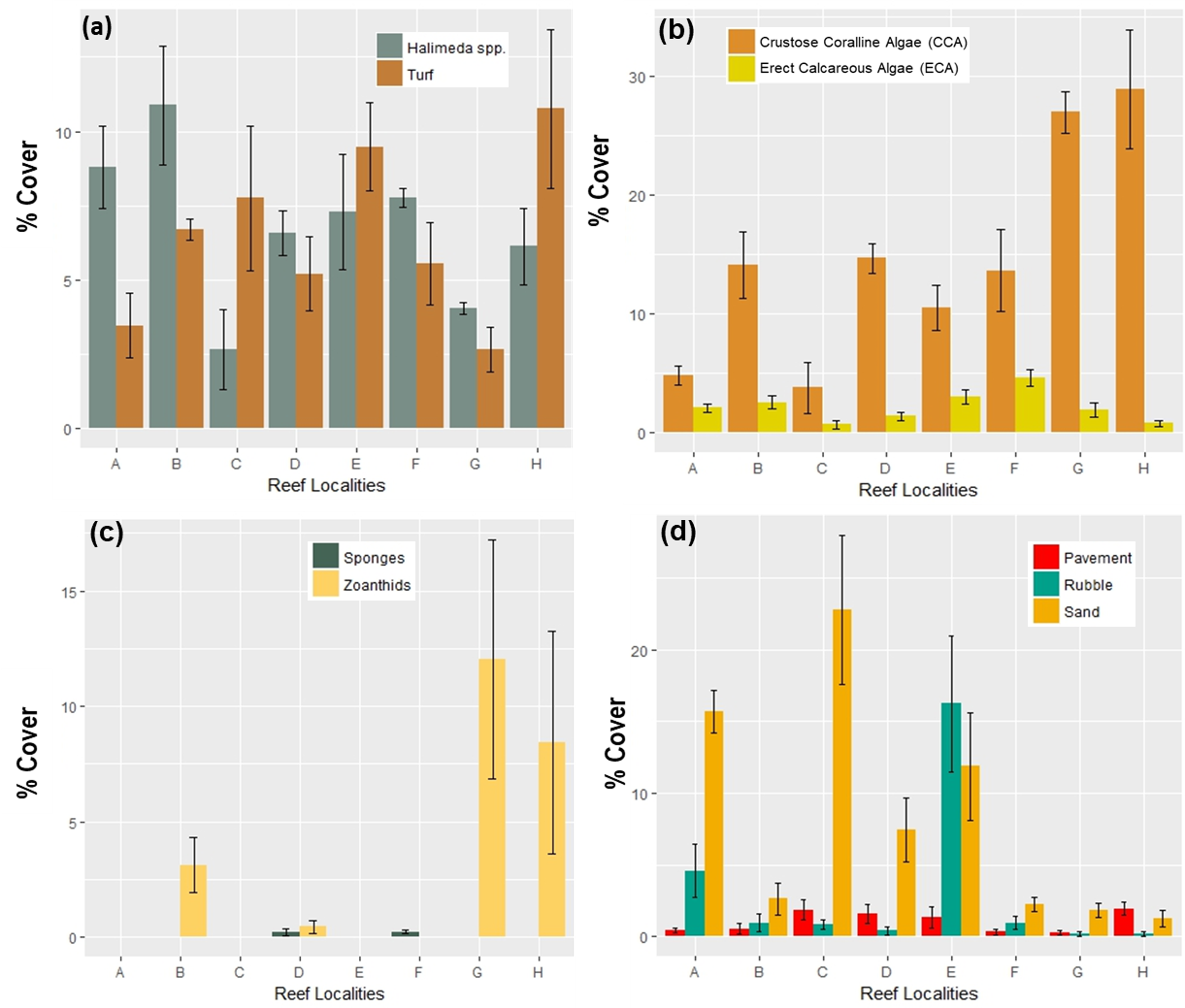

Supplement: Supplemental Information 2 — Arrow bars represent the 95% confidence intervals. Colors represent different benthic components, grouped by similar characteristics. [file peerj-08-10080-s002.png]

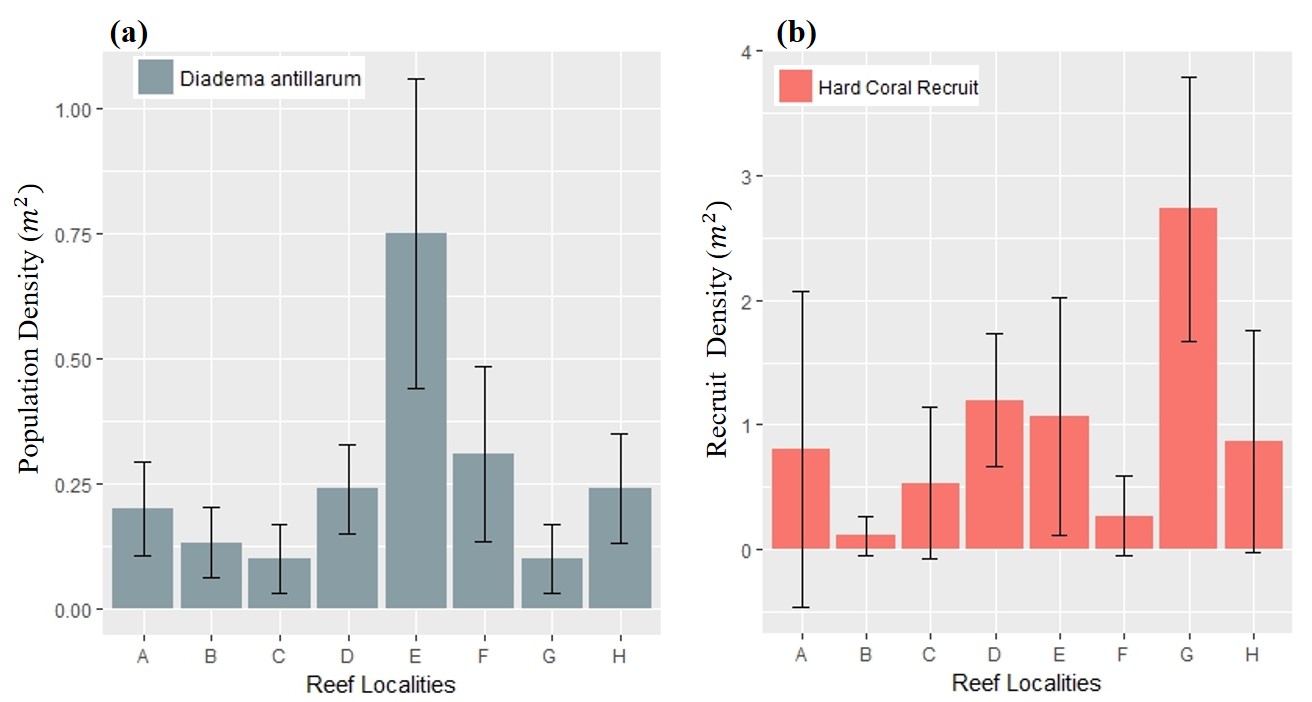

Supplement: Supplemental Information 3 — Arrow bars represent the 95% confidence intervals for five transects. (A) Mean spiny sea-urchin (Diadema antillarum) population densities, used very as a measure of reef herbivory, based on abundance counts every 20 m2 for each transect. (B) Mean sclerectinian coral recruit densities based on 0.5 m2 quadrats. [file peerj-08-10080-s003.jpg]

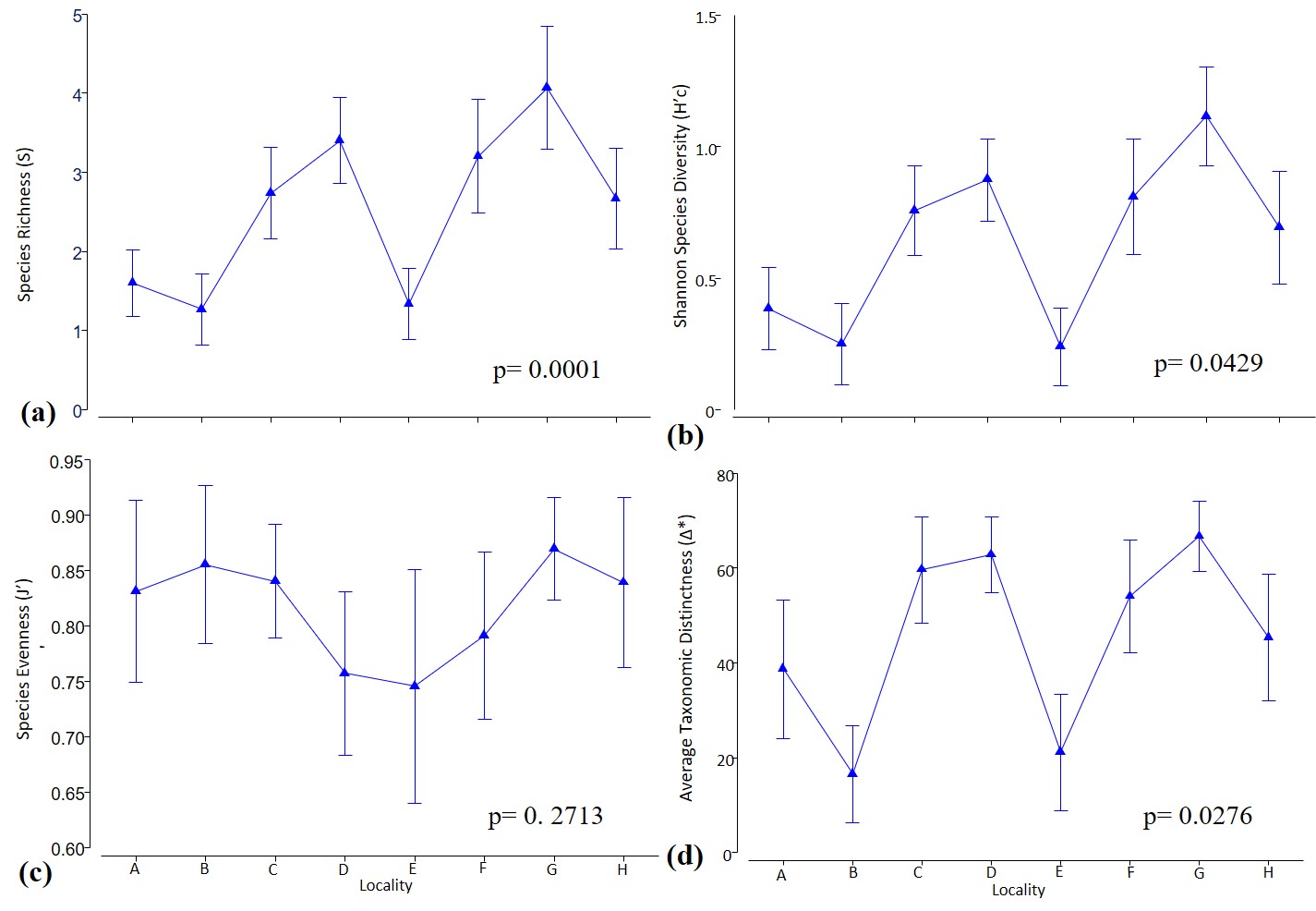

Supplement: Supplemental Information 4 — Arrow bars represent the 95% confidence intervals for five transects. P-values refer to PERMANOVA tests using each parameter as a co-variate across localities. (A) Species richness, highest at offshore sites. (B) Shannon diversity index (H’c), (C) Species evenness index (J’c) (D) Average taxonomic distinctness (Δ*). [file peerj-08-10080-s004.jpg]

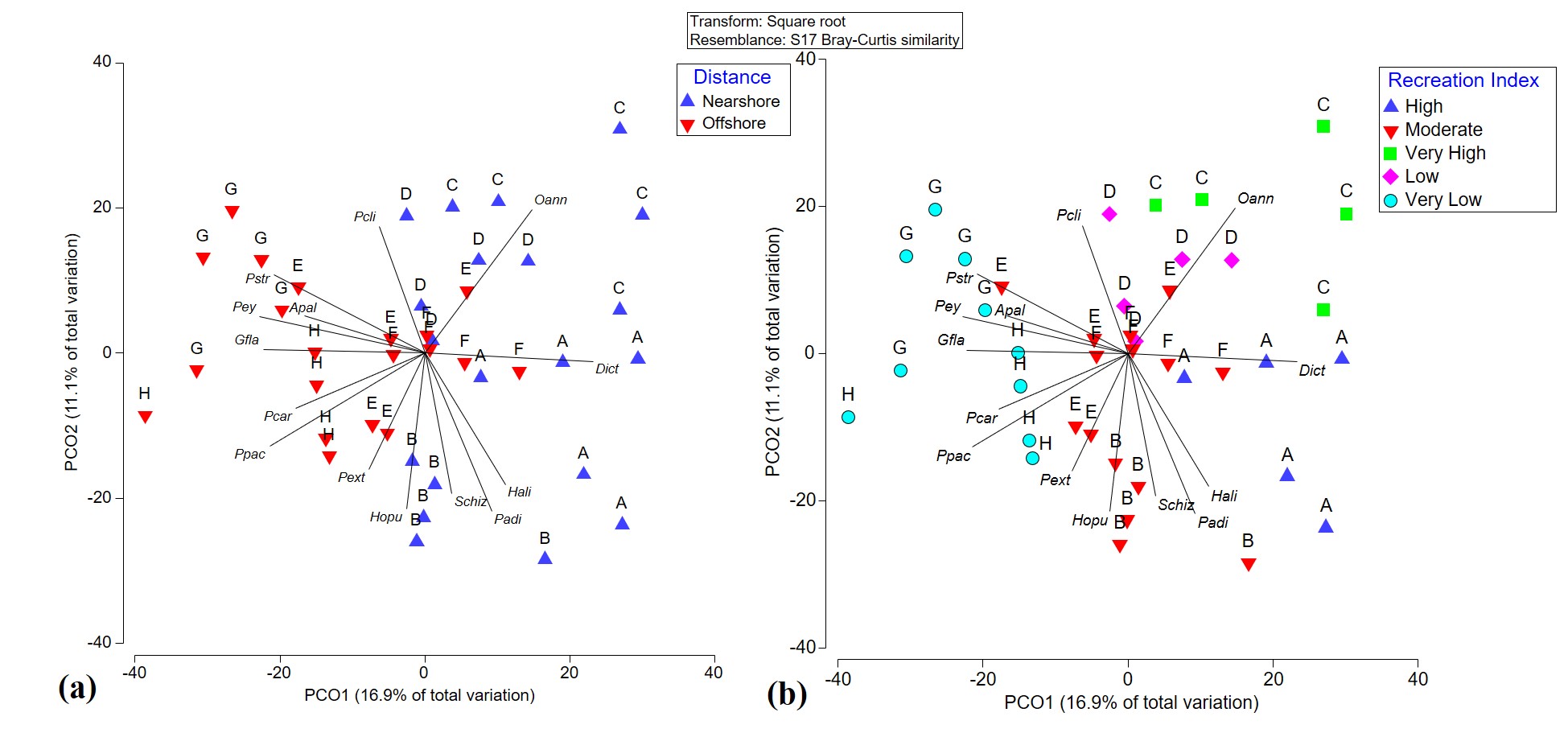

Supplement: Supplemental Information 5 — (A) Distance from the shoreline. (B) Spatial variation of the relative recreational intensity index. [file peerj-08-10080-s005.jpg]

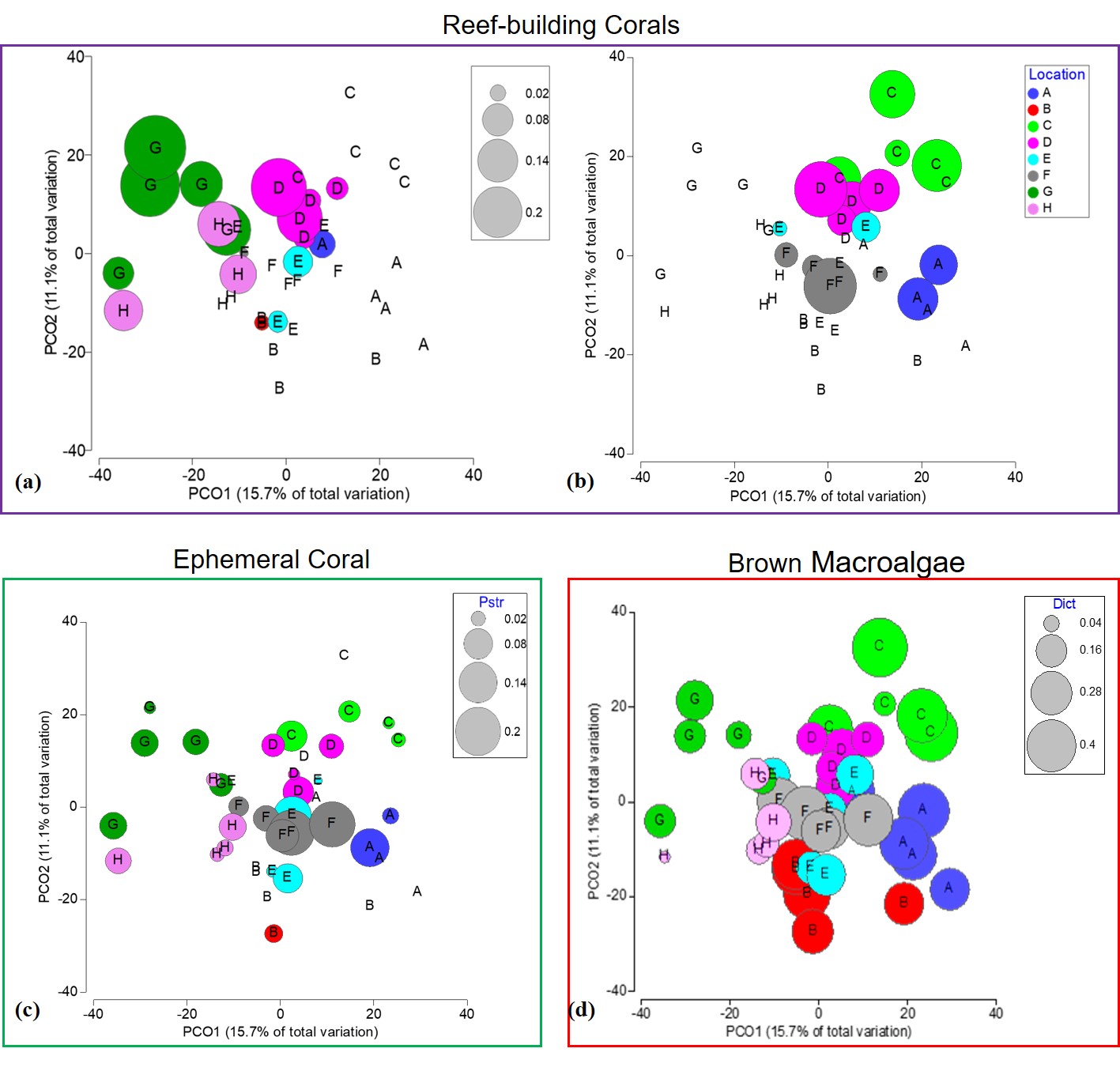

Supplement: Supplemental Information 6 — Values represent fourth root transformed counts. (A) Pseudodiploria strigosa reef-building coral species. (B) Orbicella annularis reef-building coral species. (C) Porites astreoides, a weedy coral species. (D) Dictyota spp., a common brown macroalgae genus in the Caribbean. [file peerj-08-10080-s006.jpg]

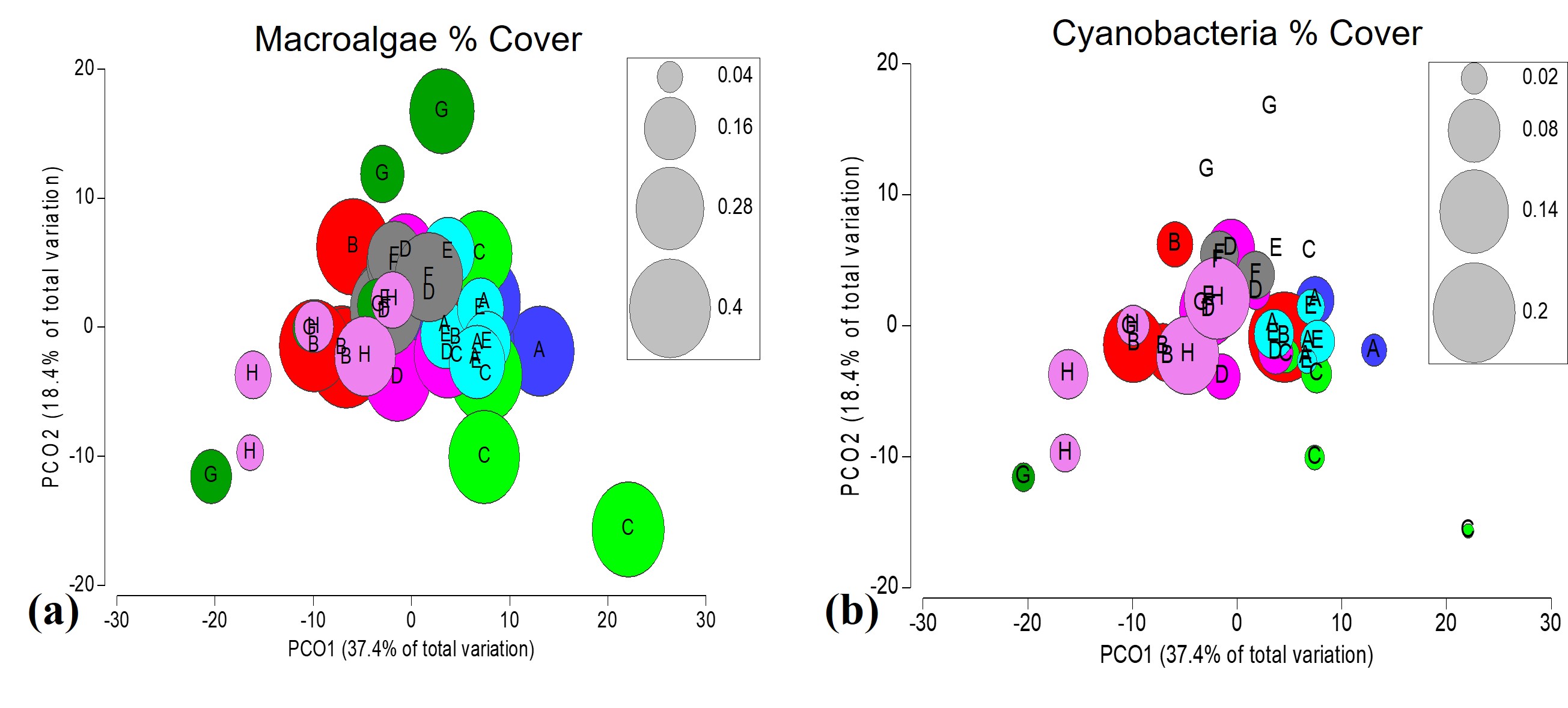

Supplement: Supplemental Information 7 — (A) Macroalgae % cover, including red, green and brown taxon showing high abundance across the study area, especially inshore locations. (B) Less abundant cyanobacteria % cover in comparison to macroalgae, but still notably present in offshore location H and inshore location B. [file peerj-08-10080-s007.jpg]

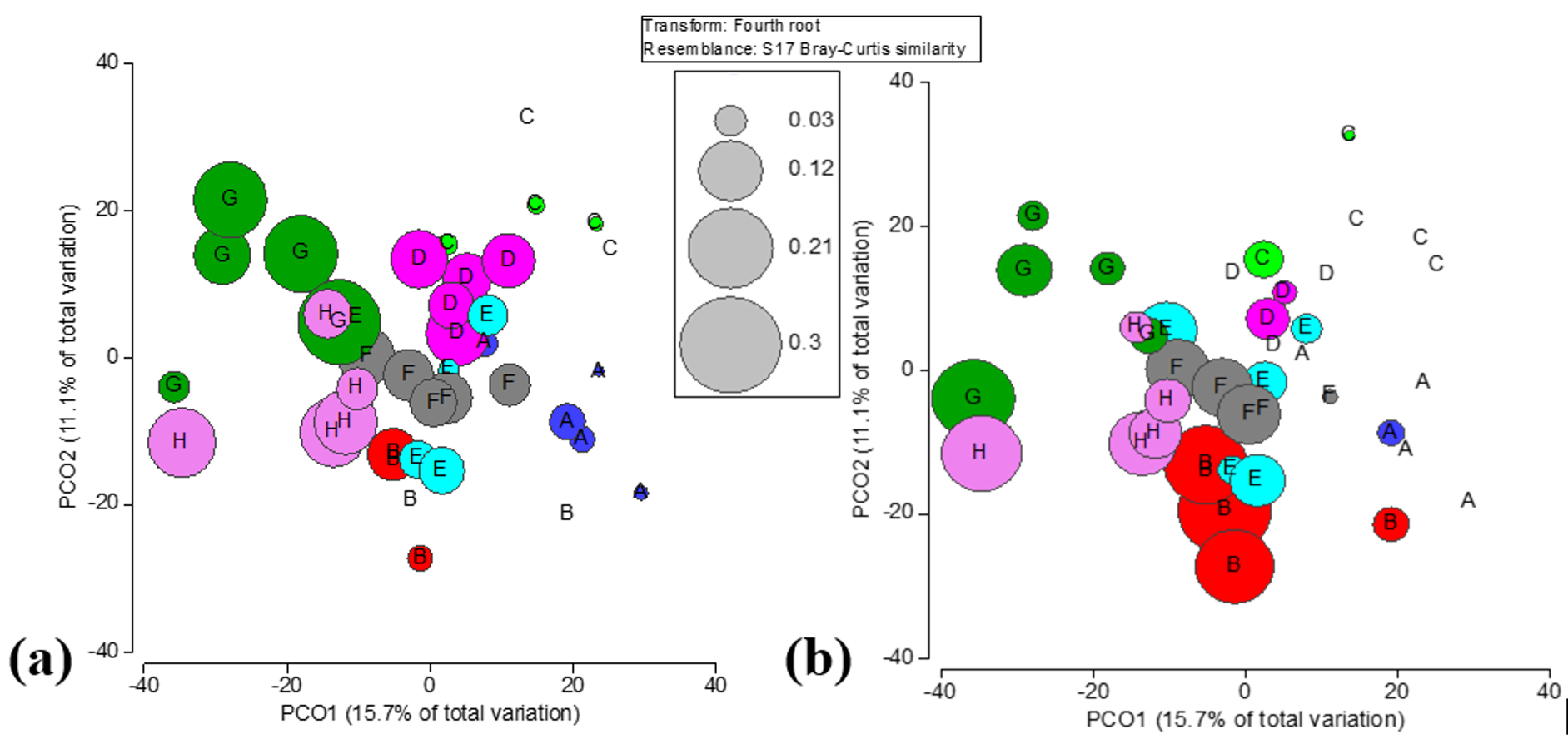

Supplement: Supplemental Information 8 — (A) Peyssonnelia spp. (B) Porolithon pachydermum. [file peerj-08-10080-s008.png]

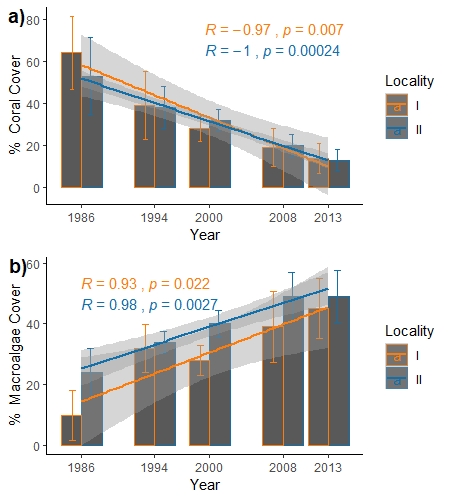

Supplement: Supplemental Information 9 — (A) Percent live cover of sclearactinian corals. (B) Percent macroalgae cover. Percent cover were based on measurements based on point counts every 0.5 m across a 10 m transect (n = 21), differing from the photo-cuadrat sampling in 2017. Arrow bars represent the 95% confidence intervals. Localities 1 and 2 correspond to the vicinity of comptemporary localitites C and D, respectively. [file peerj-08-10080-s009.jpeg]
