# Supplementary material for: Spatial benthic community analysis of shallow coral reefs to support coastal management in Culebra Island, Puerto Rico
Source: PeerJ. 2020 Oct 14;8:e10080. doi: 10.7717/peerj.10080 (PMC7568481; doi:10.7717/peerj.10080)
Supplement: Supplemental Information 10 [file peerj-08-10080-s010.docx]

**Supplementary Table 1**. Criteria to classify and interpret the measurements into indexes, according to data distributions and literature.

| **Observations** | **Classification** | | |
| --- | --- | --- | --- |
| *Rugosity Range:* | | *(Graham & Nash, 2013; Faud 2011).* |  |
| <1.25 | | Low |  |
| 1.26-1.50 | | Medium |  |
| 1.51-1.75 | | Moderately High |  |
| 1.75 | | High |  |
| *Density Range (*$m^{2})$ *from belt-transect abundances:* | | *(NEPA, 2014)* |  |
| <0.25 | | Critical |  |
| 0.25-0.49 | | Poor |  |
| 0.5-1.0 | | Fair |  |
| 1.1-2.5 | | Good |  |
| >2.5 | | Very Good |  |
| *Maximum # of aquatic recreationists during peak hours:* | | *Relative Exposure:* |  |
| 0-5 | | Very Low |  |
| 5-10 | | Low |  |
| 10-20 | | Moderate |  |
| 20-30 | | High |  |
| >30 | Very High | | |
