# Supplementary material for: Spatial benthic community analysis of shallow coral reefs to support coastal management in Culebra Island, Puerto Rico
Source: PeerJ. 2020 Oct 14;8:e10080. doi: 10.7717/peerj.10080 (PMC7568481; doi:10.7717/peerj.10080)
Supplement: Supplemental Information 11 [file peerj-08-10080-s011.docx]

**Supplementary Table 2**. Results classified according to benthic, spatial and literature categories.

| **Locality** | **Exposure to Recreational Stressors** | **Distance classification*** | **D. *antillarum***  **Index** | **Rugosity Classification for each Locality’s Transects**** | | | | |
| --- | --- | --- | --- | --- | --- | --- | --- | --- |
|  |  |  |  | 1 | 2 | 3 | 4 | 5 |
| A | High | Nearshore | Critical to Poor | M | MH | H | MH | M |
| B | Moderate | Nearshore | Critical to Poor | MH | H | H | H | H |
| C | Low | Nearshore | Critical | MH | MH | MH | MH | M |
| D | Low | Nearshore | Critical to Poor | H | H | MH | MH | H |
| E | Moderate | Offshore | Fair to Good | L | M | L | L | MH |
| F | Moderate | Offshore | Critical to Fair | MH | M | MH | MH | L |
| G | Very Low | Offshore | Critical | M | M | M | M | MH |
| H | Very Low | Offshore | Critical | M | MH | M | L | M |

*Threshold is 150 meters from the shoreline to the nearest waypoint of each locality.

** L=low; M = Medium; MH=Moderately High; H= High
