# Supplementary material for: Spatial benthic community analysis of shallow coral reefs to support coastal management in Culebra Island, Puerto Rico
Source: PeerJ. 2020 Oct 14;8:e10080. doi: 10.7717/peerj.10080 (PMC7568481; doi:10.7717/peerj.10080)
Supplement: Supplemental Information 12 — Without taking into consideration spatial variations, macroalgae exhibited the highest mean benthic cover (22.8% ± 3.0% CI), while the combination of live scleractinian and hydrocoral cover (16.7% ± 3.1%) ranked as the second most widespread cover type. This was closely followed by the presence of CCA (16.0% ± 3.4%) and later by the inorganic combination of sand, pavement and rubble (12.3% ± 3.9%), but with high variability. Octocorals (10.8% ± 2.1%) and algal turf (6.8% ± 1.7%) occupied a middle rank. However, Halimeda spp., an important genus of calcareous algae, by itself made up 6.5% ± 1.2% of the overall composition. Meanwhile, cyanobacteria, ECA, zoanthids, sponges, and other live creatures were much less abundant. The low 95% confidence intervals also suggest a sufficiently robust dataset. [file peerj-08-10080-s012.docx]

| **Supplementary Table 3.** Summary of benthic cover percent at the transect level (n=40) for all reef localities. | | | | |
| --- | --- | --- | --- | --- |
| **Cover Type** | **Mean (±SD)** | **Range** | **CI (95%)** | **SE** |
| Macroalgae | 22.86 (9.72) | 4.86 - 38.19 | 3.01 | 1.53 |
| Scleractinian and hydrocorals | 16.71 (10.05) | 1.04 - 45.83 | 3.11 | 1.59 |
| Crustose calcareous algae | 16.03 (11.04) | 0 - 38.89 | 3.42 | 1.74 |
| Octocorals | 10.82 (6.76) | 0 - 31.25 | 2.01 | 1.07 |
| Sand | 8.34 (9.78) | 0 - 42.36 | 3.03 | 1.55 |
| Turf | 6.82 (5.74) | 0 - 26.74 | 1.78 | 0.90 |
| *Halimeda sp.* | 6.51 (4.02) | 0 - 17.36 | 1.25 | 0.63 |
| Cyanobacteria | 3.49 (3.18) | 0 - 13.89 | 0.988 | 0.50 |
| Rubble | 3.35 (7.06) | 0 – 37.5 | 2.19 | 1.12 |
| Erect calcareous algae | 2.08 (1.78) | 0 - 6.94 | 0.56 | 0.28 |
| Zoanthids | 1.99 (5.86) | 0 - 28.13 | 1.82 | 0.92 |
| Pavement | 1.12 (0.44) | 0 – 4.86 | 0.44 | 0.22 |
| Other live | 0.21 (0.79) | 0 - 4.86 | 0.24 | 0.12 |
| Sponges | 0.16 (0.47) | 0 - 2.08 | 0.14 | 0.07 |
