# Supplementary material for: Spatial benthic community analysis of shallow coral reefs to support coastal management in Culebra Island, Puerto Rico
Source: PeerJ. 2020 Oct 14;8:e10080. doi: 10.7717/peerj.10080 (PMC7568481; doi:10.7717/peerj.10080)
Supplement: Supplemental Information 13 [file peerj-08-10080-s013.docx]

**Supplementary Table 4.** Comparison of highest-ranking contributions to species abundance dissimilarities between reef localities based on a SIMPER tests.

| **Highest contributing species for each interaction** | **Species Classification** | **Interacting Localities** | **Locality Dissimilarities**  **(%)** | **Species Contribution to Dissimilarities**  **(%)** |
| --- | --- | --- | --- | --- |
| *Dictyota* spp. | Brown macroalgae | A | 47.52 | 13.90 |
| *Dictyota* spp. | Brown macroalgae | B | 49.48 | 14.05 |
| *Dictyota* spp. | Brown macroalgae | C | 57.86 | 17.08 |
| *Dictyota* spp. | Brown macroalgae | D | 57.86 | 10.46 |
| *Dictyota* spp. | Brown macroalgae | E | 47.24 | 14.28 |
| *Dictyota* spp. | Brown macroalgae | F | 59.81 | 14.04 |
| *Dictyota* spp. | Brown macroalgae | G | 44.74 | 10.12 |
| *Peyssonnelia* spp, | Crustose Calcareous Algae (CCA) | H | 38.82 | 5.84 |
| *Porolithon pachydermum* | CCA | A & B | 53.98 | 5.12 |
| *Orbicella annularis* | Reef-building coral | A& C | 61.60 | 2.87 |
| *Porolithon pachydermum* | CCA | B & C | 65.95 | 3.79 |
| *Asparagopsis taxiformis* | Red macroalgae | A& D | 54.82 | 3.88 |
| *Orbicella annularis* | Reef-building massive coral | B & D | 58.91 | 3.63 |
| *Porites astreoides* | Common encrusting coral | C & D | 58.51 | 4.24 |
| *Porolithon pachydermum* | CCA | A & E | 56.49 | 4.24 |
| *Halimeda spp.* | CCA | B & E | 56.12 | 4.10 |
| *Porolithon pachydermum* | CCA | C & E | 62.10 | 3.92 |
| *Asparagopsis taxiformis* | Red macroalgae | D & E | 57.62 | 3.51 |
| *Porolithon pachydermum* | CCA | A & F | 56.05 | 3.62 |
| *Porites astreoides* | Common encrusting coral | B & F | 55.77 | 3.59 |
| *Galaxaura sp.* | Erect CCA | C & F | 62.65 | 3.18 |
| *Porites astreoides* | Common encrusting coral | D & F | 54.99 | 3.59 |
| *Eunicea succinea* | Octocoral | E & F | 51.14 | 2.97 |
| *Porites astreoides* | Common encrusting coral | A & G | 64.92 | 4.04 |
| *Porites astreoides* | Common encrusting coral | B & G | 61.29 | 4.53 |
| *Porites astreoides* | Common encrusting coral | C & G | 66.42 | 4.80 |
| *Gorgonia flabellum* | Octocoral | D & G | 57.93 | 3.84 |
| *Gorgonia flabellum* | Octocoral | E & G | 57.98 | 4.96 |
| *Porites astreoides* | Common encrusting coral | F & G | 55.78 | 4.60 |
| *Porolithon pachydermum* | CCA | A & H | 61.30 | 4.06 |
| *Padina spp.* | Brown macroalgae | B & H | 50.89 | 4.35 |
| *Orbicella annularis* | Reef-building massive coral | C & H | 64.06 | 3.68 |
| *Orbicella annularis* | Reef-building massive coral | D & H | 51.47 | 4.09 |
| *Gorgonia flabellum* | Octocoral | E & H | 56.64 | 4.79 |
| *Gorgonia flabellum* | Octocoral | F & H | 54.97 | 3.64 |
| *Pseudolithoderma extensum* | CCA | G & H | 51.44 | 4.20 |
