# Supplementary material for: Spatial benthic community analysis of shallow coral reefs to support coastal management in Culebra Island, Puerto Rico
Source: PeerJ. 2020 Oct 14;8:e10080. doi: 10.7717/peerj.10080 (PMC7568481; doi:10.7717/peerj.10080)
Supplement: Supplemental Information 14 [file peerj-08-10080-s014.docx]

| **Supplementary Table 5.** PERMANOVA analysis of coral biodiversity (H’c, 57 species) in response to environmental co-variates, plus distance and locality terms. | | | | | | | | | |
| --- | --- | --- | --- | --- | --- | --- | --- | --- | --- |
| **Source** | **d.f.** | **SS** | | **MS** | | **Pseudo-F** | | **P (perm)** | |
| Rugosity Index | 1 | 136.27 | 136.27 | | 3.171 | | 0.0949 | |  |
| Diadema Density | 1 | 12.818 | 12.818 | | 0.26661 | | 0.7202 | |  |
| Coral Recruitment Density | 1 | 64.249 | 64.249 | | 1.4094 | | 0.2627 | |  |
| % Diseases | 1 | 990.59 | 990.59 | | 17.797 | | **0.0002** | |  |
| S | 1 | 4201 | 4201 | | 84.664 | | **0.0001** | |  |
| H'c | 1 | 123.58 | 123.58 | | 2.1067 | | **0.0429** | |  |
| J'c | 1 | 72.658 | 72.658 | | 1.2804 | | 0.2713 | |  |
| AvTD | 1 | 249.4 | 249.4 | | 4.562 | | **0.0276** | |  |
| VarTD | 1 | 54.943 | 54.943 | | 0.96743 | | 0.361 | |  |
| # of Visitors | 1 | 21.347 | 21.347 | | 0.77675 | | 0.5201 | |  |
| Distance | 1 | 30.928 | 30.928 | | 0.68365 | | 0.5231 | |  |
| Locality (Distance) | 5 | 202.45 | 40.489 | | 0.63309 | | 0.7437 | |  |
| **Res** | 23 | 1471 | 63.955 | | - | | - | |  |
| **Total** | 39 | 7631.2 | - | | - | | - | |  |
| \| Pseudo F statistics were calculated for each term using direct analogues to univariate expectations of mean squares. A sequential Type I sums of squares was used. P-values were obtained using 9999 permutations under a reduced model, with each term contributing either a fixed or random component to the overall model. \| \| --- \| | | | | | | | | | |
