# Supplementary material for: Spatial benthic community analysis of shallow coral reefs to support coastal management in Culebra Island, Puerto Rico
Source: PeerJ. 2020 Oct 14;8:e10080. doi: 10.7717/peerj.10080 (PMC7568481; doi:10.7717/peerj.10080)
Supplement: Supplemental Information 15 — The default weighting exponential (α) for IDW interpolations is 2. However, we did not assume that the distance-decay relationship is constant over space, and adjusted this power value according to the nearest neighbor statistic in order to increase spatial predictive accuracy in our clustered samples, as recommended by Lu et al. (2008). Therefore, the α was adjusted to optimize the cross validation of predicted values at unknown location by both maximining the regression function between expected and observed values and minimizing its root mean squared error (RMSE). [file peerj-08-10080-s015.docx]

| **Supplementary Table 6:** Cross-validation results determining the best power value for Inverse Distance Weighting interpolations of ecological parameters. | | | |
| --- | --- | --- | --- |
| **Parameter** | **Power (α)** | $\boldsymbol{R}^{\boldsymbol{2}}$ | **RMSE** |
| Coral richness | 4.66 | ‎0.88 | 1.97 |
| Coral diversity | 4.55 | ‎0.88 | 0.19 |
| Coral disease abundance | 4.73 | ‎0.86 | 0.04 |
| Coral recruit density | 6.53 | ‎0.98 | 0.26 |
| % Live coral cover | 4.45 | ‎0.86 | 5.99 |
| % Macroalgae cover | 2.98 | ‎0.86 | 5.16 |
| % Cyanobacteria cover | 4.85 | ‎0.93 | 1.25 |
| % CCA cover | 5.05 | ‎0.92 | 4.02 |
| 1. *cervicornis* abundance | 2.5 | ‎0.80 | 0.18 |
| 1. *palmata* abundance | 4.58 | ‎0.94 | 0.78 |
| 1. *antillarum* abudance | 7.72 | ‎0.99 | 0.06 |
| Rugosity | 7.53 | ‎0.97 | 0.06 |
