# Supplementary material for: Spatial benthic community analysis of shallow coral reefs to support coastal management in Culebra Island, Puerto Rico
Source: PeerJ. 2020 Oct 14;8:e10080. doi: 10.7717/peerj.10080 (PMC7568481; doi:10.7717/peerj.10080)
Supplement: Supplemental Information 16 — The CRRI is composed of fifteen indicators grouped in to the Coral Index (% coral cover, bleaching, species richness and recruitment density), threatened species (A. palmata, A. cervicornis, O. annularis, O. faveolata) and Algal Index (macroalgae, turf, CCA, Halimada spp., Dictyota spp. Lobophora spp., Ramicrusta/Peyssonnelia spp.). [file peerj-08-10080-s016.docx]

| **Supplementary Table 7:** Summary of site-averaged results for the Coral Reef Resilience Index (CRRI) for Flamenco Bay. The CRRI is composed of fifteen indicators grouped in to the Coral Index (% coral cover, bleaching, species richness and recruitment density), threatened species (*A. palmata, A. cervicornis, O. annularis, O. faveolata)* and Algal Index ( macroalgae, turf, CCA, *Halimada spp., Dictyota spp. Lobophora variegata, Ramicrusta/Peyssonnelia*). | | | | |
| --- | --- | --- | --- | --- |
| **Location** | **Global CRRI** | **Coral Index** | **Threatened Species Index** | **Algal Index** |
| A | 2.49 (poor) | 2.75 | 1 (critical) | 3.71 (good) |
| B | 2.55 (poor) | 2.50 | 1 (critical) | 4.14 (good) |
| C | 2.57 (poor) | 3.00 | 1 (critical) | 3.71 (good) |
| D | 2.93 (fair) | 3.50 | 1.25 (critical) | 4.28 (very good) |
| E | 2.68 (fair) | 2.50 | 1 (critical) | 4.28 (very good) |
| F | 2.67 (fair) | 3.00 | 1 (critical) | 4 (good) |
| G | 2.81 (fair) | 3.00 | 1 (critical) | 4.42 (very good) |
| H | 2.73 (fair) | 2.75 | 1 (critical) | 4.42 (very good) |
